# Nanotechnology-Based Therapeutics for Airway Inflammatory Diseases

**DOI:** 10.1007/s12016-024-09019-w

**Published:** 2025-02-10

**Authors:** Limei Cui, Yujuan Yang, Yan Hao, Hongfei Zhao, Yu Zhang, Tong Wu, Xicheng Song

**Affiliations:** 1 Department of Otolaryngology, Head and Neck Surgery, Qingdao Medical College, Qingdao University, Yantai Yuhuangding Hospital, Qingdao University, Yantai, 264000 China; 2 Shandong Provincial Key Laboratory of Neuroimmune Interaction and Regulation, Yantai, 264000 China; 3 Shandong Provincial Clinical Research Center for Otorhinolaryngologic Diseases, Yantai, 264000 China; 4 Shandong University of Traditional Chinese Medicine, Jinan, 250000 Shandong China; 5 Medical Research Center, The Affiliated Hospital of Qingdao University, Qingdao University, Qingdao, 266000 China

**Keywords:** Airway inflammatory diseases, Nano-delivery systems, Traditional drug delivery, RNA drug delivery, Immunotherapy drug delivery

## Abstract

Under the concept of “one airway, one disease”, upper and lower airway inflammatory diseases share similar pathogenic mechanisms and are collectively referred to as airway inflammatory diseases. With industrial development and environmental changes, the incidence of these diseases has gradually increased. Traditional treatments, including glucocorticoids, antihistamines, and bronchodilators, have alleviated much of the discomfort experienced by patients. However, conventional drug delivery routes have inherent flaws, such as significant side effects, irritation of the respiratory mucosa, and issues related to drug deactivation. In recent years, nanomaterials have emerged as excellent carriers for drug delivery and are being increasingly utilized in the treatment of airway inflammatory diseases. These materials not only optimize the delivery of traditional medications but also facilitate the administration of various new drugs that target novel pathways, thereby enhancing the treatment outcomes of inflammatory diseases. This study reviews the latest research on nano-drug delivery systems used in the treatment of airway inflammatory diseases, covering traditional drugs, immunotherapy drugs, antimicrobial drugs, plant-derived drugs, and RNA drugs. The challenges involved in developing nano-delivery systems for these diseases are discussed, along with a future outlook. This review offers new insights that researchers can utilize to advance further research into the clinical application of nano-drug delivery systems for treating airway inflammatory diseases.

## Introduction

The respiratory tract is the pathway by which air travels in and out of alveoli and consists of the nose, pharynx, larynx, trachea, bronchi, and the tubes before the terminal bronchioles. Clinically, the nose, pharynx, and larynx are usually referred to as the upper airway, and the trachea, main bronchi, and all levels of bronchi in the lungs comprise the lower airway. In 1997, Grossman [1] proposed the “one airway, one disease” concept, which pointed out that the upper and lower airways constituted a complete respiratory pathway system and were related in pathogenesis, pathophysiology, and anatomy. With changes in the environment and gradual ageing of the world’s population, the incidence of airway inflammatory diseases such as allergic rhinitis (AR), chronic sinusitis (CRS), asthma, and chronic obstructive pulmonary disease (COPD) has continued to increase.

Currently, corticosteroids, leukotriene regulators, histamine regulators, bronchodilators, and specific immune methods are commonly used in the clinical treatment of airway inflammatory diseases. However, traditional drug treatments and immunotherapy do not always provide acceptable outcomes due to a variety of physiological barriers within the airway. One such barrier is a mechanical barrier, which occurs when an inflammatory condition of the airway leads to mucous membrane swelling or excessive mucus secretion, which subsequently leads to respiratory stenosis. Additionally, epithelial cell cilia movement can remove drugs deposited in the airway. Other types of barriers include chemical and immune barriers, which mainly consist of surfactants, proteolytic enzymes, and macrophages. Researchers are currently attempting to overcome the adverse effects of these barriers, which would be helpful for improving the efficacy of drugs used to treat airway inflammatory diseases.

Nanotechnology refers to the manipulation of atomic and molecular particles that are usually 10–100 nm in diameter. Nanomedicine, as an interdisciplinary research field, has received extensive worldwide attention from researchers [2]. Nanotechnology has a variety of applications, including the development of new methods for drug delivery, vaccine development, medical testing, disease diagnosis, and the manufacture of drug formulations used in personalized medicine [3]. Nano-carrier systems for drug delivery can bypass physiological barriers, increase the solubility and accessibility of a drug in the body, control drug release, prolong the drug release time, and reduce the dose of a drug to lower its adverse effects. These unique advantages will greatly change the way we practice medical treatment.

Nano-drug delivery technology has been investigated for the diagnosis and treatment of airway inflammatory diseases. Numerous excellent reviews have previously elucidated the potential applications of nanomaterials in the treatment of lower airway diseases; however, there is a lack of discussions that integrate both upper and lower airway considerations. This review primarily focuses on the application of nano-drug delivery strategies in traditional pharmaceuticals and novel therapeutic approaches. It aims to provide innovative insights for researchers in airway disease, nanotechnology, and clinical practitioners, thereby advancing the technological progress in drug delivery for airway inflammatory diseases.

## Pathophysiology of Airway Inflammatory Diseases

### Allergic Immune Response

**Fig. 1:**
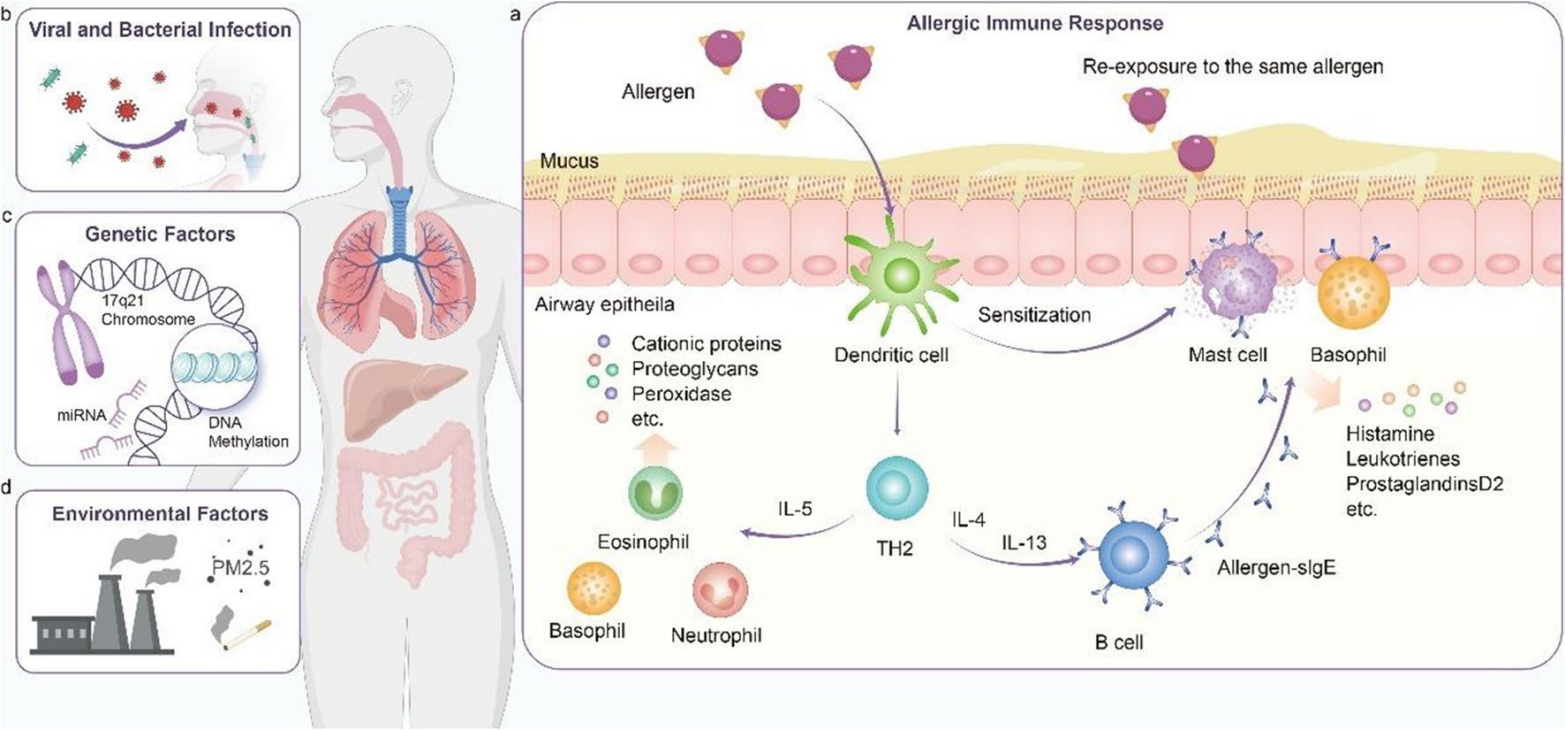
The schematic representation of the pathophysiology of airway inflammatory diseases. **a** Allergic immune response; **b** viral and bacterial infections; **c** genetic factors; **d** environmental factors

The pathogenesis of airway inflammatory diseases is multifaceted. Allergic reactions play a significant role as key etiological factors that contribute to the development of airway inflammation diseases such as AR, CRS, and allergic asthma. Pollen, organic dust, fungal spores, and animal dander penetrate the body via the nasal and bronchial epithelium. These allergens are subsequently identified by dendritic cells (DCs) within the airway mucosa and presented to naïve T cells. This interaction prompts the differentiation of naïve T cells into T-helper 2 (TH2) cells, which secrete TH2-associated cytokines. These cytokines in turn activate B cells, leading to the production of immunoglobulin E (IgE) [4, 5]. Upon re-exposure to the same allergen, IgE cross-links with FcεRI receptors, leading to the activation and degranulation of mast cells and eosinophils. Upon re-exposure to the same allergen, IgE cross-links with FcεRI receptors, leading to the activation and degranulation of mast cells and eosinophils. Mast cell release of histamine, leukotrienes, prostaglandin D2, and other mediators which subsequently induce responses in nasal nerves, blood vessels, and glands [6]. Activated eosinophils not only release leukotrienes and inflammatory mediators but also secrete major basic protein (MBP) and eosinophil cationic protein (ECP), which can induce damage to epithelial cells [7]. These proteins, in conjunction with other chemokines, facilitate the recruitment of neutrophils and macrophages to participate in the inflammatory response. Macrophages play a pivotal role throughout the allergic response; they can phagocytose allergens and present antigens to T cells, while also modulating the differentiation of T cells toward the TH2 phenotype through the release of inflammatory mediators [7, 8]. TH2 cell-mediated immune response is also critically involved. Upon entry into cells, allergens are identified and activate the allergen-specific TH2 cells, leading to the production of substantial quantities of interleukin-4 (IL-4), interleukin-5 (IL-5), and interleukin-13 (IL-13) cytokines. These cytokines further enhance vascular permeability, facilitate eosinophil infiltration into the nasal mucosa, and increase mucus production. Consequently, these processes culminate in producing various airway disease phenotypes, including nasal congestion, rhinorrhea, cough, and chest tightness [9, 10]. This process is illustrated in Fig. 1a.

### Non-allergic Mechanism

#### Viral and Bacterial Infection

Viral and bacterial infections play a significant role in contributing to airway inflammatory diseases (Fig. 1b). In children, asthma attacks are primarily triggered by the respiratory syncytial virus and human rhinovirus [11]. In adults, the influenza virus and metapneumovirus are also major contributors [12]. *Streptococcus pneumoniae*, *Haemophilus influenzae*, and *Moraxella catarrhalis* are the main bacteria that cause respiratory infections, and *H. influenzae* is particularly important in managing stable asthma. Other bacterial infections can also increase the frequency of acute asthma attacks. Viruses and bacteria are present in approximately 50% of severe and 25% of moderate COPD exacerbations [13, 14].

#### Genetic Factors

Genetic factors can increase the risk for airway inflammatory diseases, with some allergic conditions sharing genetic susceptibilities (Fig. 1c). A 2007 genome-wide association study (GWAS) identified a locus on chromosome 17q21 linked to increased asthma susceptibility in children [15]. Japanese researchers then employed linkage disequilibrium mapping in two separate populations and confirmed that variants in that locus were associated with AR [16]. These overlapping sites may help explain the “allergic match” and “one airway, one disease” concepts for AR and asthma. The interaction between genetic risk and factors such as smoking is a key area of research. Findings indicate that epigenetic mechanisms, such as DNA methylation and microRNA expression, play a role in airway inflammatory diseases. For example, differential methylation sites associated with COPD are enriched in proximity to loci identified by the GWAS as being related to the disease [17]. Epigenetic modifications result in prolonged suppression or activation of gene expression following exposure to risk factors, with the changes accumulating over time. Such mechanisms may elucidate why early exposure to risk factors contributes to the development of airway inflammatory diseases in later stages of life.

#### Environmental Factors

In modern society, rapid economic development and accelerated urbanization have made the impact of environmental pollution on airway inflammatory diseases increasingly prominent. Among the primary harmful factors are fine particulate matter, such as PM2.5, and exposure to tobacco smoke [18]. The adverse effects induced by PM2.5 are primarily due to the size of the particles and the toxic substances adsorbed onto them. PM2.5 exposure can disrupt the balance of T cell differentiation and regulate airway inflammatory responses through pathways such as the aromatic hydrocarbon receptor (AhR) and toll-like receptors (TLR) 2/TLR4/Myd88, leading to epithelial cell damage and inflammatory cell infiltration [19]. Research has also shown that PM2.5 can induce mitochondrial rupture and dysfunction in human airway epithelial cells [20]. Animal studies involving mice exposed to PM2.5 under specific germ-free conditions demonstrated increased inflammatory cell infiltration in the airways, which can be mitigated by effective interventions [21].

Smoking is a major risk factor for the development of COPD and is significantly associated with the onset of asthma [22–24]. Tobacco contains numerous harmful substances, including nicotine, tar, and carbon monoxide, which affect epithelial-mesenchymal transition (EMT) and damage airway epithelial cells via pathways such as STAT, thereby disrupting cellular structure and function [25]. These substances also stimulate immune cells in the respiratory tract, such as macrophages and neutrophils, prompting the release of various inflammatory mediators. This leads to airway inflammation, mucosal edema, congestion, increased mucus secretion, and airway narrowing. Additionally, inflammatory mediators can induce airway smooth muscle contraction, increasing airway resistance and causing symptoms such as dyspnea [26].

### Nano-drug Delivery Systems for Airway Inflammatory Disorder

Drug therapy is currently the principal method for quickly, effectively, and safely controlling nasal, tracheal, or pulmonary symptoms in patients with airway inflammation. The most commonly used first-line medications for allergic airway inflammatory diseases include nasal glucocorticoids, antihistamines, and leukotriene receptor antagonists. For lower airway diseases, bronchodilators and adrenergic receptor agonists are also used. Traditional medications reach high initial concentrations in the body; however, they cannot effectively target the affected sites and require frequent administration. Moreover, they also irritate the respiratory mucosa. When airway inflammation occurs, there is an increase in mucus secretion, which leads to airway narrowing. It has been observed that the mechanical and mucus barriers of the airways significantly impede the efficacy of traditional drug treatments in reaching and being absorbed at the site of inflammation. This inefficacy results in higher drug concentrations, which in turn cause damage and irritation to the airway mucosa [27]. Furthermore, a substantial portion of the administered drug is diverted to the kidneys, stomach, and other organs, leading to adverse reactions. At the same time, the stability of pharmacological agents, including antigens and peptides administered as immunotherapy, is compromised by degradation.

The rapid development of nanotechnology offers hope for developing controlled and sustained drug delivery and release systems [28]. Depending on the nanomaterials employed, nano-drug delivery systems can be categorized into several types, including lipidic nanoparticles such as liposomes [29], polymeric nanoparticles such as PLGAs [30], inorganic nanoparticles such as mesoporous silica nanoparticles (MSNs) [31], metal nanoparticles such as Au [32], and hydrogels such as chitosan [33]. A nano-drug delivery system, which utilizes nanoparticles as carriers, offers significant advantages including high biocompatibility, controlled and sustained drug release, and enhanced drug stability. For instance, the pharmacokinetic parameters of orally administered free docetaxel and docetaxel-loaded chitosan nanomicelles indicate a significant increase in Cmax by threefold with the nanomicelle formulation, achieving peak concentration in a quarter of the time compared to the free drug. The relative bioavailability is enhanced by 2.5 times [34]. Nanotechnology offers promising opportunities by targeting key cells involved in inflammatory responses [35]. Alveolar macrophages (AM) are pivotal effectors in the inflammatory processes of the lower airways. Liposomes modified with hyaluronic acid (HA) and loaded with dexamethasone exhibit significantly improved targeting efficiency toward AM compared to control liposomes lacking the drug. Moreover, this targeted strategy leads to an approximate 30% reduction in the expression of M1-type inflammatory factors [36].

**Fig. 2:**
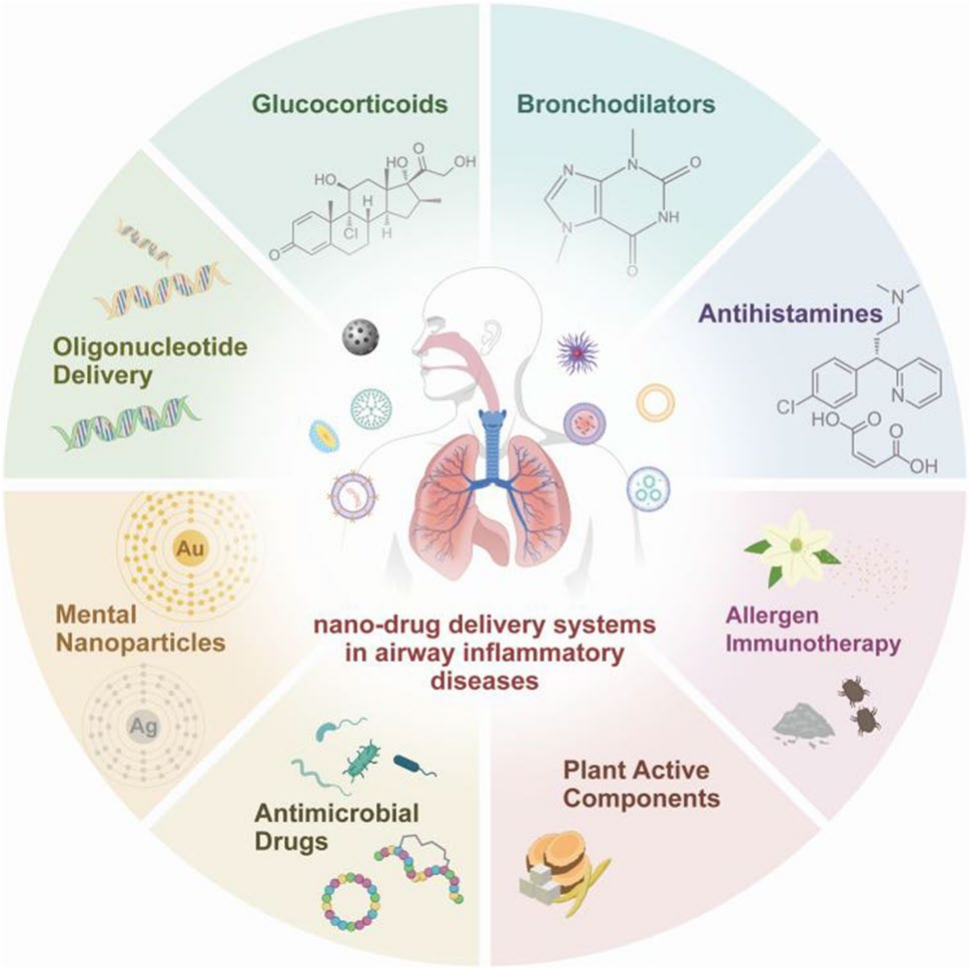
Drug classes delivered by nanoparticle systems in airway inflammatory diseases

Furthermore, these systems can undergo various surface modifications to enhance drug targeting efficiency. Typically, surface-modified nanomaterials can release drugs slowly or in a controlled manner in response to local microenvironmental conditions, such as particular temperature, low pH, and high level of reactive oxygen species [37]. Additionally, modified nanoparticles can effectively target specific cells, such as epithelial cells, macrophages, and eosinophils [38–40], allowing for selective drug release, improved drug utilization, and reduced systemic toxicity. Recent advancements have catalyzed an extensive investigation into nano-drug delivery systems in airway inflammatory diseases. These systems have enhanced the efficacy of delivering conventional therapeutic agents (e.g., glucocorticoids, antihistamines, and allergens) directly to airway tissues; additionally, nucleotide-based drugs, previously deemed undeliverable, can now be administered more stably via nano-drug delivery mechanisms (Fig. 2).

## Strategy for Using a Nano-Drug Delivery System in Airway Inflammatory Diseases

### *Nanoparticles for Glucocorticoid Delivery Please check if the section headings are assigned to appropriate levels*

Glucocorticoids, such as budesonide and ciclesonide, play a crucial role in the treatment of allergic airway inflammatory diseases. These steroids are typically lipophilic, which allows them to enter cells and exert their biological effects by binding to glucocorticoid receptors in the airway mucosa [41]. This binding initiates the transcription of anti-inflammatory genes and suppresses the expression of pro-inflammatory genes. A locally acting nasal delivery system of triamcinolone acetonide (TA) has been developed for the maintenance therapy of AR. Studies have encapsulated TA in three different nano-carriers: polymeric oil core nanocapsules (NCs), lipid nanocarriers such as nanoemulsion (NE), and nanostructured lipid carriers (NLCs) to investigate their effects on mucosal permeation, retention, and intranasal deposition in vivo. It was found that NCs produced least mucosal irritability and enhanced the stability of TA compared to the other two nano-carriers. In vivo studies on nasal cavity deposition indicated that NCs maintained the drug in the nasal mucosa for the longest duration and reduced side effects due to systemic absorption [42].

Budesonide is a commonly used glucocorticoid. Many researchers optimize NP characteristics for optimum drug deposition and pharmacokinetics in the airways [43–45]. Nanoparticles have been discovered to be carriers capable of increasing a drug’s biophysical and chemical stability [46, 47]. An intranasal administration of budesonide-loaded lipid-core nanocapsule (BudNCs) was explored by Manoel et al. to treat asthma combined with AR. When compared to commercial budesonide, the BudNCs were more efficient in reducing immune cell influx and restraining airway remodeling, and evidence gathered with a long-term asthma model was more compelling than evidence gathered with a short-term model [48].

### Nanoparticles for Antihistamine Delivery

Histamine enhances antigen-presenting capacity, triggers the release of inflammatory mediators from mast cells and basophils, and promotes the chemotaxis of eosinophils and neutrophils. Histamine (H1) receptors, which are expressed in various cells, including neurons and vascular smooth muscles, regulate vasodilation and bronchoconstriction, and are closely associated with Type I hypersensitivity reactions[49]. H1 receptor antagonists, also known as H1 antihistamines, exert their pharmacological effects by reversibly competing for the H1 receptor, and directly or indirectly influencing the expression of inflammatory factors and cell mediators [41]. Cetirizine is an antihistamine commonly used for treating allergic airway diseases, but its hydrophobic nature and irritation to the respiratory mucosa limit its application. Several studies have explored its nano-delivery. Mirella Mirankó used hydroxypropyl methylcellulose (HPMC) combined with Miglyol 812 to create a composite oleogel carrying cetirizine, which provided local anti-swelling effects[50]. Simultaneously, they also employed nano spray drying techniques to prepare various adhesive polymers and cyclodextrin complexes to enhance the penetration of cetirizine into the nasal mucosa [51]. Sun et al. covalently bound cetirizine to hydroxybutyl chitosan polysaccharide and deoxycholic acid to create CDHBC nanoparticles that release cetirizine in the specific physiological environment of the nasal cavity (pH 5.5, 33 °C, with the presence of lysozyme) [52]. They also encapsulated the H1 antagonists, cetirizine and ketotifen, in hydroxybutyl chitosan nanoparticles for combined medication to further inhibit the release of histamine [53].

### Nanoparticles for Bronchodilator Delivery

Salbutamol, a typical short-acting β2-adrenergic receptor agonist, reduces airway spasms by effectively inhibiting the release of mediators such as histamine [54]. Nagaraja and others utilized a nano spray drying mechanism to prepare salbutamol-loaded poly (lactic-co-glycolic acid)-polyethylene glycol (PLGA-PEG) microspheres (SPPs). In vivo results indicated that the salbutamol-loaded PLGA-PEG SPP formulation achieved high targeting efficacy and drug concentrations in the lungs, along with good safety [55]. Incorporating salbutamol sulfate into liposomes promoted the diffusion of salbutamol sulfate and extended in vitro drug release by > 90% [56].

### Nanoparticles for Allergen Delivery

Specific immunotherapy (SIT) is an available treatment option for patients with AR and allergic asthma. The mechanism of SIT is based on inducing immune tolerance; inhibiting the homing and tissue migration of Th2 cells producing IL-4, IL-5, and IL-13; and reducing the numbers and mediators of basophils and mast cells [57, 58]. SIT involves administering allergens at continuous low doses by various methods, including injection or inoculation through the skin or mucosal surface [59]. Clinically, dust mites and pollen are the primary allergens used for immunotherapy and can be administered subcutaneously or sublingually. A large retrospective cohort study showed that patients undergoing allergen immunotherapy (AIT) exhibited significantly reduced asthma controller and medication use when compared to placebo-treated patients, indicating improved asthma control and a reduced likelihood of asthma exacerbations [60]. A current challenge with SIT is the inefficient delivery of allergens to target dendritic cells. Therefore, developing formulations with smaller and more effective doses of allergens is a critical area of research. PLGA is suitable for peptide or protein formulations, as it protects them from degradation, provides for high drug loading rates, and allows for slow drug release [61, 62]. It has been used in allergen-specific immunotherapy in recent years. When pollen allergen chea3 was loaded into PLGA of different molecular weights, the Th2 immune responses in AR mice were attenuated and shifted toward TH1 and Treg immune responses [63].

Recent studies have focused on using various delivery systems and adjuvants to develop new formulations that enhance the efficacy of intranasal immunotherapy (INIT). Hu et al. created a DNA vaccine by co-expressing the dust mite protein component Der p 2 and tumor necrosis factor-alpha-induced protein 3 (TNFAIP3) and then encapsulating them into PLGA nanoparticles for sublingual injection. TNFAIP3, also known as A20, regulates the function of various immune cells and is involved in maintaining immune balance. This DNA vaccine alleviated nasal allergic inflammatory reactions in mice with AR and promoted an increase in splenic Treg cells in the AR mice [64].

### Nanoparticles Loaded with Plant Active Components

Natural medicinal materials are used for treating various diseases due to their bioactive components. Numerous studies have been performed on the therapeutic use of plant active components, such as baicalin, curcumin, ginsenosides, andrographolide, and angelica gigas in treatment of airway inflammatory diseases [65–68]. However, these types of agents often have poor solubility, are subject to the first-pass effect, and are chemically unstable. Researchers have found that optimizing drug delivery methods by the use of nanotechnology can enhance the therapeutic effects of these drugs [69]. Baicalin, a herbal extract with multiple pharmacological effects on respiratory diseases [70–72], has been delivered by use of nanotechnology techniques such as liposomes, nano-emulsions, micelles, phospholipid complexes, and solid dispersions [65, 73]. Chitosan, known for its mucoadhesive properties and permeation enhancement, is widely used in sustained drug release systems. Wang et al. loaded baicalin onto chitosan and found that it was effective in controlling the pathophysiology of asthma [74]. Moreover, in a mouse model of acute lung injury, liposome-loaded baicalin was superior to free baicalin for reducing the wet/dry ratio, reducing lung injury scores, decreasing pro-inflammatory cytokines (TNFα, IL-1β), and reducing the total protein in bronchoalveolar lavage fluid (BALF) [75]. Curcumin, the active component of turmeric, demonstrated anti-inflammatory effects in diseases such as asthma and AR. Encapsulation in nanosomes enhanced the therapeutic effectiveness of curcumin in asthma treatment, and lipid-loaded curcumin also more effectively reduced inflammation markers in asthma [76, 77]. Sanza [78] encapsulated curcumin and ovalbumin (OVA) in PLGA nanoparticles for treatment of AR and found that it enhanced the efficiency of immunotherapy. Thus, it is evident that the future of research on plant active components in airway inflammatory diseases will increasingly enter the nanotechnology era.

### Nanoparticles Loaded with Antimicrobial Drugs

Bacterial infections are a significant cause of persistent and refractory CRS and COPD. Bacterial resistance and the side effects of long-term use limit the efficacy of conventional antibiotics, leading to a rapidly increasing proportion of infections that cannot be treated with standard antibiotics. Therefore, the development of new antimicrobial and anti-inflammatory treatments is urgently needed. Colloidal silver, also known as nanosilver, consists of metallic particles ranging from 10 to 100 nm in diameter. Research indicates that nanosilver can serve as a biologically active adjuvant to antibiotics and help to kill *Staphylococcus aureus* (including MRSA) and *Pseudomonas aeruginosa* biofilm cells both in vitro and in vivo, thus playing a therapeutic role in CRS and COPD [79–81]. However, when used in conjunction with different antibiotics, silver nanoparticles can enhance the resistance of clinically isolated strains. Furthermore, individual silver nanoparticles are prone to oxidation and/or aggregation in air, which hinders their practical application. Seracin, serving as a reducer, dispersant, and stabilizer for synthesized silver nanoparticles, promotes re-epithelialization. Researchers have reacted sericin with silver nitrate to synthesize the green product SF-NS [82]. A subsequent study clearly demonstrated that the SF-NS solution significantly inhibited the formation of mature biofilms by *Staphylococcus aureus* in vivo in a concentration-dependent manner, with subsequent recovery of epithelia and cilia after treatment with SF-NS solution [83]. Bhuvanesh et al. reported a composite of silver nanoparticles (AgNPs) and silk-elastin-like protein-based polymers (SELP) that showed high biocompatibility with human nasal epithelial cells. The composite allowed for the sustained release of AgNPs and silver ions over 3 days and exhibited effective antibacterial activity against *Pseudomonas aeruginosa* and *Staphylococcus aureus* in vitro [84]. Despite multiple studies assessing the safety of colloidal silver both in vitro and in vivo, clinical considerations regarding the required concentration for efficacy, cellular uptake, and potential toxic effects of silver nanoparticles remain to be addressed.

Other antibacterial nanomaterials have been the subject of extensive research, with antibacterial peptides and peptide-based composite nanomaterials representing a significant subset in this field. Antimicrobial peptides (AMPs) have garnered considerable attention due to their ability not only to eradicate bacteria but also to modulate host responses. They exhibit low affinity for mammalian cell membranes and minimal toxicity, while being effective against antibiotic-resistant bacterial strains. However, the delivery of AMPs is complex due to their large size, positive charge, and amphipathic nature. Therefore, AMP delivery systems have recently come into focus. Biomaterial delivery can maintain AMPs at sufficiently high concentrations for prolonged periods, producing significant antimicrobial effects while also protecting the AMPs from degradation. Additionally, shielding AMPs reduces exposure to non-target tissues and cells, thus diminishing associated toxicity. Yu et al. studied MSNs loaded with AMPs, melittin and ofloxacin, and then covered with cyclodextrin or adamantane, and found they had enhanced anti-biofilm activity. The host–guest interaction between adamantane and cyclodextrin led to the formation of co-assemblies that included melittin and ofloxacin, resulting in a drug combination with superior bactericidal capabilities [85]. Notably, Yang et al. developed a multifunctional gold/silver nanocage with AMPs and hyaluronic acid (HA). The nanometal components enabled phototherapy, the AMPs provided enhanced targeting and bactericidal effects, and HA suppressed immune responses while providing a non-toxic coating for the nanometal particles. This composition, when exposed to near-infrared light, significantly reduced bacterial presence in a mouse model of pulmonary inflammation [86].

A novel mucolytic and biofilm-penetrating immunomodulatory antimicrobial agent (IMAM) has been developed for the nebulized treatment of COPD. The IMAM consists of hollow mesoporous silica nanoparticles (HMSN) encapsulating ceftazidime (CAZ), with side chains bearing both positively charged quaternary ammonium and negatively charged carboxyl groups. At neutral pH, flexible, randomly coiled peptides accumulate on the surface of the HMSN and covers its pores to prevent CAZ leakage. In a COPD mouse model, the negatively charged IMAM effectively penetrated mucus and biofilm due to electrostatic repulsion with glycoproteins and polysaccharides. In the moderately acidic environment of the biofilm, the peptides are conditionally activated by the acid-triggered removal of their carboxyl groups, and transformed into positively charged rigid α-helices capable of disrupting bacterial membranes while concurrently releasing CAZ. Thus, the helical antimicrobial peptides synergistically act with CAZ to eradicate colonized bacteria. Moreover, the positively charged peptides can effectively clear bacterial extracellular DNA (eDNA) and genomic DNA (gDNA) that inhibits the infiltration and activation of immune cells [87].

To fully leverage the potential of antimicrobial nanomaterials, further work is needed to link drug delivery aspects with the broader biological effects of such materials. Many aspects of antimicrobial nanomaterials remain to be explored, including their immunomodulatory roles, recruitment of leukocytes and monocyte/macrophages, modulation of neutrophil responses, promotion of neutrophil extracellular traps, impact on antigen-specific adaptive immunity, and effects on the microbiome with regard to mucosal immunity.

### Metallic Nanoparticles

Metallic nanomaterials not only have robust antimicrobial properties but also exhibit anti-inflammatory effects, which allows them to play a role in the treatment of airway inflammatory diseases [88, 89]. As early as the 2010s, studies on gold and silver nanoparticles demonstrated their significant potential as therapeutic agents. For example, Emiliano et al. reported that intranasal administration of gold nanoparticles at 1 h before ovalbumin challenge could inhibit 70–100% of allergen-induced inflammatory cell accumulation and pro-inflammatory factor production, as well as reduce oxidative stress, and ultimately prevent key pathological changes in an asthma mouse model [90]. Similarly, Magda, et al. found that nebulized gold nanoparticles (AuNPs) produced efficacious outcomes in a corticosteroid-resistant asthma mouse model by enhancing the levels of HDAC2 and NRF2 expression [91]. Silver nanoparticles have also been found to mitigate allergic airway inflammation, potentially due to downregulation of the NFκB pathway and PI3K/HIF-1α/VEGF signaling pathway [92–94].

Metal nanoparticles can also serve as drug carriers; for example, gold chloride mixed with a caffeoylxanthiazonoside (CFX) solution extracted from Xanthium strumarium was formulated into spherical CFX-coated gold nanoparticles. When administered to an AR mouse model, the gold nanoparticles were found to reduce nasal inflammatory cell infiltration and inhibit the production of Th2 cytokines. However, the specific mechanisms of these nanoparticles were not detailed, and the therapeutic role of CFX-coated gold nanoparticles requires further investigation [95]. There are also concerns about the safety of metal nanomaterials. Studies have found that low doses of inhaled AuNPs can lead to bronchial interstitial inflammation, exacerbate lung inflammation, and have adverse health effects [96–98]. Therefore, it is crucial to repeatedly evaluate how manufactured metal nanoparticles affect the structure and function of the mucosal barrier in animal models before conducting human trials [99].

### Nanoparticles for Oligonucleotide Delivery

In recent years, RNA-based therapies have gained widespread attention for use in treating various diseases due to their significant therapeutic effects and high specificity [100]. Lung diseases offer a variety of currently undruggable but attractive targets that could potentially be treated with RNA drugs. Many types of RNA drugs are currently under investigation, including antisense oligonucleotides (ASOs), small interfering RNAs (siRNAs), microRNAs (miRNAs), and aptamers. However, free RNA molecules are easily degraded and cannot be effectively delivered to disease sites. The full therapeutic potential of RNA is gradually being realized by using various nanocarrier delivery systems to protect RNA molecules at lower concentrations and improve their clinical effects. Dendrimeric polymers, lipid-based nanoparticles, targeted specific ligand-oligonucleotide conjugates, and antibody conjugates are excellent options for enhancing the efficiency of oligonucleotide delivery [101–103]. The use of oligonucleotides in treatment of respiratory diseases is a significant advancement, and the selection of targets is crucial.

An imbalance in the differentiation ratios of TH1, TH2, and TREG cells is a fundamental cause of inflammation, with an increase in TH2 differentiation leading to airway inflammation and hyper-reactivity. Thus, it is particularly important to investigate genes involved in regulation of T cell differentiation. IFN-γ is a multifunctional TH1 cytokine that can downregulate the expression of TH2-related cytokines. Kumar et al. formulated IFN-γ plasmid DNA, encapsulated it in chitosan, and delivered it intranasally to a mouse model of allergic asthma. The results indicated that a reduction in IFN-γ degradation and a significant suppression of TH2 cytokines (IL-4 and IL-5) also alleviated airway inflammation and hyper-reactivity [104]. Xie and others innovatively developed an siRNA delivery system that targets activated T cells. The system was based on a ligand-polymer conjugate (transferrin-polyethyleneimine [Tf-PEI]) and exploited the increased expression of transferrin receptors on activated T cells. TfR, a membrane protein, is expressed at low levels on most cell surfaces, but can rapidly bind and become internalized upon recognition of the iron transport glycoprotein, Tf. Fluorescently labeled siRNA revealed that the Tf-PEI conjugate system could effectively target and deliver siRNA into activated T cells. In vitro experiments with primary human activated T cells or Jurkat cells showed significant knockdown of the target gene. Although the system could be taken up by activated T cells in vivo, its release after internalization was not satisfactory [105]. Further improvements were made by the team using melittin-PEI to enhance endosomal escape. They designed effective sequences targeting the classic TH2 cytokine GATA3 for knockdown. This delivery system successfully delivered GATA3 siRNA to pulmonary epithelia in lung explants and achieved a knockdown efficiency of 3%. Subsequent ex vivo tests performed using precision-cut human lung slices demonstrating good safety and provided support for the translational potential of these research findings [106].

The activation of NFκB plays a crucial role in the pathogenesis of airway inflammatory diseases, notably by enhancing the airway infiltration of neutrophils and activating inflammatory signaling cascades in respiratory epithelia. Consequently, researchers are highly interested in developing inhibitors of NFκB activation, as summarized in several comprehensive reviews [107, 108]. Early studies demonstrated that NFκB oligodeoxynucleotides (ODNs) could reduce the expression of inflammatory cytokines both in vivo and in vitro, and the efficient targeted delivery of those ODNs has become a focal point of research. The use of nanoparticles such as PLGA, PEI, and liposomes to carry NFκB decoy ODNs in airway diseases has been extensively reported. The use of nanoparticles to load ODNs significantly enhanced the duration of the inhibitory effect, thereby effectively reduced the levels of IL-6, IL-8, and mucin-2, as well as other factors [109]. Liposomes, in particular, can protect decoy ODNs from degradation by nucleases and extend the circulating half-life of the ODNs[110]. Wijagkanalan et al. took advantage of the ability of macrophage surface mannose receptors to recognize mannose and fucose and synthesized a mannose-cationic liposome delivery system for NFκB decoy ODNs. When compared to naked decoy ODNs, this composite delivery system primarily targeted alveolar macrophages and inhibited the activation of NFκB, thereby suppressing the release of TNF-α, IL-1β, CINC-1, and the infiltration of neutrophils [111]. Moreover, the inclusion of adjuvants such as cholesterol and fish protamine in liposomes can further optimize the delivery of NFκB decoy ODNs within airways [112].

Thymic stromal lymphopoietin (TSLP) is a cytokine secreted by airway epithelial cells (AECs) in response to various environmental insults. It plays a pivotal role in mediating the interface between the body and environmental stimuli and in orchestrating the TH2 immune response. Lipid nanoparticles (LNPs) loaded with siRNA were utilized to specifically knock down TSLP expression in stimulated AECs. This study leveraged the characteristic increase in Intercellular Adhesion Molecule-1 (ICAM-1) expression in AECs under conditions of asthma inflammatory stimulation in order to enhance the specific delivery efficiency of siRNA into AECs. A cyclic peptide resembling the rhinovirus coat protein, which specifically binds to ICAM-1, was conjugated to the LNPs, which were subsequently loaded with TSLP siRNA to form a PEP-LNPs-siTSLP nanosystem. This system delivered TSLP siRNA specifically to AECs which showed high cellular TSLP uptake. Treatment with TSLP siRNA reduced the secretion of pro-inflammatory factors, effectively alleviated inflammatory pathological manifestations, and demonstrated good safety and efficacy [113].

Vitamin D-binding protein (VDBP) is a major pathogenic factor in allergic asthma, as demonstrated by proteomic analyses of bronchoalveolar lavage fluid. Under inflammatory conditions, VDBP expression increases in alveolar macrophages and bronchial epithelial cells. Reducing the concentration of VDBP protein was found to alleviate respiratory inflammation in a mouse model of asthma [114]. Choi et al. developed a DEXA-PEI/VDBP siRNA system that loaded dexamethasone and VDBP siRNA simultaneously using PEI. The results showed that VDBP siRNA was detectable in 20% of the cells in bronchoalveolar lavage fluid and 30% of lung tissue epithelial cells. When compared to a DEXA-PEI group, the DEXA-PEI/VDBP siRNA group showed a 10% reduction in the number of inflammatory cells. This novel combined treatment strategy reduced the dosage of dexamethasone, thereby lowering its side effects [115].

MiRNAs, such as miRNA 21, miRNA 146a, and miRNA 155, play a crucial role in respiratory diseases by silencing target gene expression via degradation or translational inhibition. MiRNA-based therapies have become a practical approach for the clinical treatment of airway inflammatory diseases [116, 117]. MiR-146a targets *IRAK1*, *TRAF6*, and *STAT5B* and other genes to regulate immune responses and suppress inflammation, particularly in allergic airway inflammatory diseases [118]. Currently, miR146a has received great attention in the field of nano-delivery research. Su et al. used PEG-PLA nanoparticles to deliver miR-146a for treatment of AR. Those investigators developed a gel/NPs/miR-146a binary formulation that demonstrated improved nucleic acid delivery capability and positive pharmacodynamic effects in a rat model [119]. Adel et al. adsorbed miR-146a onto nanoparticles composed of polyglycerol sebacate-co-ω-pentadecalactone (PGA-co-PDL) and cationic lipid dioleoyl trimethylammonium propane (DOTAP) to reduce the expression of a target gene, *IRAK1*. In vitro release profiles of the miR-146a-loaded nanoparticles showed continuous release up to 77% after 24 h, and a 40% reduction in *IRAK1* expression. An increase in miR-146a expression in the airways has been observed in the context of Rhinovirus (RV) infection-induced airway inflammation and asthma [120]. Treatment with a cell-penetrating peptide (CPP)-miR-146a nanocomplex not only prevented the increase in pro-inflammatory chemokines caused by an RV infection but also reduced the inflammatory response in a mouse model of allergic airway inflammation induced by house dust mite (HDM) extract [121].

Generally, RNA therapeutics exert a significant influence on airway inflammatory diseases, predominantly by modulating T cell differentiation and suppressing the expression of inflammatory factors. While these therapeutic agents show considerable potential for future application, their delivery systems must be enhanced to optimize their effects.

### Delivery Routes of Nanomedicines in Airway Inflammatory Diseases

Due to the unique structure of the airways, medications for airway inflammatory diseases are typically delivered locally via nasal sprays, inhalation (including dry powder inhalers [DPIs] and metered-dose inhalers [MDIs]), and nebulization rather than orally, the majority of them form aerosols that facilitate the delivery of medication to the upper or lower airways during device propulsion or inhalation [122]. Each of these localized delivery methods has distinct characteristics in terms of drug delivery efficiency and device functionality (Table 1).

**Table 1:** Different routes of administration for nano-drug delivery in the airway

Delivery routes	Overall advantages	Advantages	Challenges
Intranasal	• Minimal invasive nature • Targeted delivery • Avoidance of first-pass metabolism • Rapid onset of action	• Avoidance of first-pass metabolism • Ease of use • Good patient compliance	• Limited volume capacity • Variability in absorption • Potential for irritation • Limited formulation options
Nebulized		• Various delivery devices (soft mist or dry powder inhalers) • Direct delivery to the trachea and lungs • Suitable for all age groups • Deliver high doses of medication • Ideal for drugs that cannot be formulated as powders	• High demand may lead to medication wastage • Microbial residue caused by reusable devices
Inhaled		• Portable and compact • Adjustable medication doses • Longer shelf life	• High oropharyngeal deposition rate • Limited properties of the medication

The nasal cavity, with its large absorptive surface area, rich capillary network, and low local enzymatic activity, is ideal for drug absorption [123]. Nasal administration is a non-invasive method that bypasses hepatic first-pass metabolism, allowing for rapid drug absorption. It is commonly used to treat upper airway inflammatory diseases such as AR and CRS [124]. When using nasal nano-delivery systems, it is important to consider the mucociliary clearance mechanisms that may resist drug absorption. Additionally, potential local irritation to the nasal mucosa from the drug or its excipients should be minimized. Nasal delivery is also constrained by volume and molecular weight limitations, typically ranging from 25 to 200 µL and not exceeding 1 kDa [125].

For lower airway inflammatory diseases such as asthma and COPD, inhalers are commonly used to deliver nanocarrier systems, targeting affected lung tissue with inhaled nanoaerosols to enhance therapeutic efficiency. Pulmonary delivery bypasses hepatic first-pass metabolism, providing an advantageous environment for drug bioavailability. The respiratory system’s large surface area and the thin, vascularized alveolar epithelium facilitate both local and systemic drug delivery [126, 127]. Inhalation delivers high drug concentrations directly to the target site while maintaining low systemic levels, thus minimizing exposure to other organs and reducing side effects. Additionally, inhalation can enhance drug bioavailability in the lungs and potentially achieve a relatively uniform drug distribution within the alveoli [128]. Inhalation devices that deliver drugs directly into the lungs require specific techniques for effective use.

In clinical practice and research, nebulization is also a common method, converting liquid medication into smaller droplets for inhalation. However, this method often results in significant drug loss and requires longer administration time. To date, the inefficient delivery of nanomaterials to the airways remains a significant challenge, indicating substantial room for improvement in the modification of nano-delivery systems and delivery devices [122, 125, 129].

## Current Challenges in Using Nano-Delivery Systems in Treatment of Airway Inflammatory Diseases

**Fig. 3:**
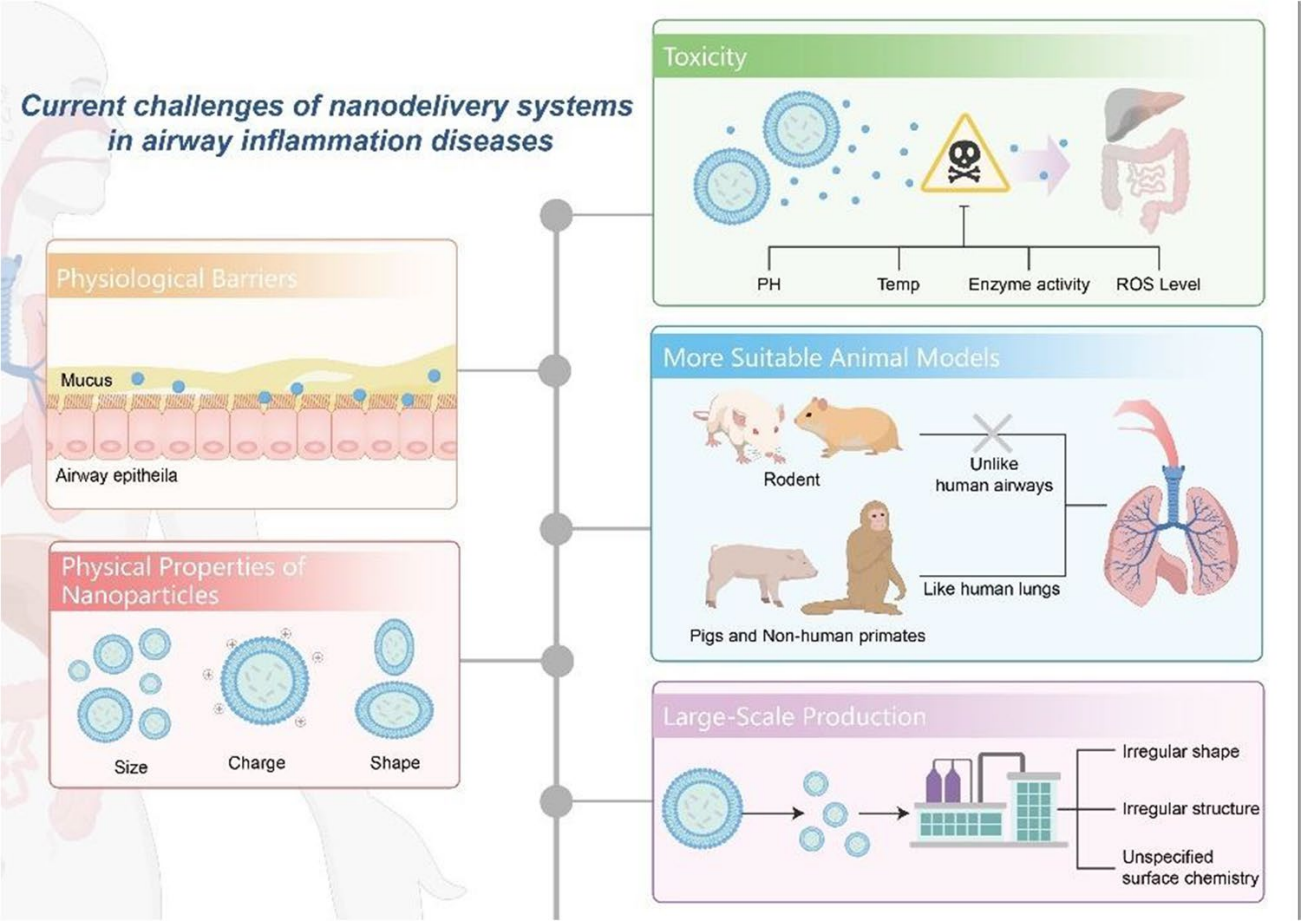
Current challenges of nano-delivery systems in airway  inflammatory diseases

Although nanocarriers have become a most promising strategy for drug delivery, but still are in early stages. Currently, there is limited research on the clinical registration of nanomedicines for treating airway inflammatory diseases, particularly those affecting the upper airway conditions. Table 2 summarizes the nano-drugs that entered clinical trials. The high attrition rate in the later stages of drug development underscores the lack of innovation in new drug development [138]. Nano-delivery systems face significant challenges both in achieving laboratory success and in transitioning from the lab to clinical applications for airway inflammatory diseases, as illustrated schematically in Fig. 3.

**Table 2:** Nanomedicine clinical trials for airway inflammatory diseases (registered at https://clinicaltrials.gov/)

Approval	Particle type	Therapeutic agent	Indication	Brief description	Delivery routes	Status	Administration dosage	Last update posted	Reference
NCT01315678	Liposomal	Amikacin	CF patients due to *Pseudomonas aeruginosa*	Determine whether Arikayce™ is effective in treating chronic lung infections caused by Pa in CF participants compared with Tobramycin TOBI	Nebulization inhalation	Phase III	590 mg of Arikace™ foradministered for three cycles (28 days on-treatment followed by 28 days off-treatment)	2020.06.16	[130]
NCT01316276	Liposomal	Amikacin	CF patients due to *Pseudomonas aeruginosa*	Assess the prolonged safety and tolerability of a daily 590 mg dose of liposomal amikacin for inhalation (LAI) in CF patients with persistent *Pseudomonas aeruginosa* infection	Nebulization inhalation	Phase III	590 mg of Arikace™ for 2 years	2020.06.17	[131]
NCT03038178	Liposomal	Amikacin	CF patients due to *Pseudomonas aeruginosa*	Assess the efficacy, safety and tolerability of liposomal-amikacin for inhalation (LAI) plus standard of care (SOC) mycobacterial multi-drug regimen for treatment of mycobacterium abscessus lung disease	Nebulization inhalation	Phase II	590 mg of Arikace™ for 12 months	2020.03.04	/
NCT00777296	Liposomal	Amikacin	CF patients due to *Pseudomonas aeruginosa*	Multidose safety and tolerability study of dose escalation of liposomal amikacin for inhalation (ARIKACE™) in CF patients with chronic infections due to *Pseudomonas aeruginosa*	Nebulization inhalation	Phase II	280 mg, and 560 mg of Arikace™ for 28 days	2020.07.30	[132, 133]
NCT00558844	Liposomal	Amikacin	CF patients due to *Pseudomonas aeruginosa*	Determine the safety and tolerability of different does Arikayce™ in CF patients infected *Pseudomonas aeruginosa*	Nebulization inhalation	Phase I/II	70 mg, 140 mg, and 560 mg of Arikayce™ for 28 days	2019.06.24	[132, 133]
NCT02344004	Liposomal	Amikacin	Nontuberculous mycobacterial (NTM) lung infection due to Mycobacterium avium complex (MAC)	Evaluate the effectiveness of LAI to multi-drug regimen (MDR) in participants with NTM lung infection caused by MAC that were refractory to treatment	Nebulization inhalation	Phase III	590 mg Arikace™ + MDR for up to 19 months	2020.05.07	[134]
NCT01315236	Liposomal	Amikacin	Refractory nontuberculous mycobacteria (NTM) lung disease	Evaluate the efficacy, safety and tolerability of LAI in patients with treatment of NTM lung disease	Nebulization inhalation	Phase II	590 mg of Arikayce™ for 84 days	2019.08.21	[135]
NCT02081963	Liposomal	Amikacin	Acute exacerbation of non-cystic fibrosis bronchiectasis	Evaluate the efficacy, indications and adverse reactions associated with nebulized amikacin administration	Nebulization inhalation	Phase IV	200 mg Arikace™ twice a day for 14 days	2019.04.24	/
NCT05712538	Lipid nanoparticles	CFTR mRNA	CF	Determine the safety, tolerability and pharmacokinetics of ARCT-032 in healthy adult subjects (Phase 1) and CF patients (Phase 1b)	Nebulization inhalation	Phase I	Single dose of ARCT-032 for up to 2 weeks	2024.10.15	/
NCT03375047	LNP	cystic fibrosis transmembrane conductance regulator (CFTR) mRNA	CF	Evaluate the safety and tolerability of single and multiple escalating doses of MRT5005; the first inhaled mRNA therapeutic	Nebulization inhalation	Phase I/II	Single and multiple escalating doses of MRT5005	2020.11.16	[136]
NCT01621867	Lipid vector	cystic fibrosis transmembrane conductance regulator (CFTR) gene	CF	Test the clinical efficacy, safety, and tolerability of repeated nebulized doses of a gene product coding for a normal CFTR protein	Nasal application/nebulization inhalation	Phase II	12 doses of nebulized gene therapy at intervals of 4 weeks over a 48-week period	2015.10.22	[137]
NCT03574805	Lipocalin-1	IL-4Ra antagonist (PRS-060)	Asthma	Investigate the safety, tolerability, and pharmacokinetics of multiple doses of PRS-060	Oral inhalation	Phase I	Multiple doses of PRS-060	2020.12.24	/
NCT03059017	Nano-vesicles	salbutamol sulfate	asthma	Study the relative bioavailability of different salbutamol sulphate inhaler formulations in healthy male subjects	Oral inhalation	Phase I	0.8 mg for one time	2017.02.23	/

### Challenges of Preclinical Studies

#### Physiological Barriers

Airways possess complex inherent defense mechanisms, such as intricate mechanical barriers, as well as chemical and immune barriers within the airway microenvironment that limit particle entry and prevent the inhalation of pathogens and irritants. These barriers also pose significant challenges for effective nasal drug administration. Under inflammatory conditions, the nasal and bronchial mucosa secrete excess mucin that creates a thick mucus layer. This mucus exhibits hydrophobic, electrostatic, and hydrogen bonding interactions and adheres to particles. Additionally, cross-linked mucin fibers create a dense porous structure which forms a barrier that drugs must penetrate for effective cellular delivery. Zhang et al. recently found that siRNA-LNPs could be effectively delivered to approximately 45% of respiratory epithelial cells; however, < 5% of endothelial cells showed LNP uptake. Moreover, siRNA fluorescence visualization of lung tissue sections indicated that siRNA fluorescence along the bronchial epithelial cells was approximately two-fold that observed in the lower respiratory tract and alveoli [113]. This reminds us that, despite promising findings in nano-drug delivery for airway inflammatory diseases, the efficiency of that delivery method remains low. Methods for designing mucus-penetrating nanoparticles or utilizing mucus-inert polymers are urgently needed, such as altering the surface functional groups of nanoparticles (e.g., hyaluronic acid–based modifications), charge, and particle size can significantly impact their performance [139, 140]. Meanwhile, expanding the mucosal lattice spacing by disrupting specific non-covalent interactions within the mucus gel using mucolytic agent-modified and protein hydrolase-based modified nanoparticles is an effective strategy to enhance mucosal drug penetration [141]. In the future, it is imperative to design additional nanoparticles that are small, hydrophilic, and net neutral to effectively inhibit adhesion.

#### Physical Properties of Nanoparticles

Research has shown that the size, shape, and charge of nanoparticles play crucial roles in their cellular uptake by airway mucosal surfaces, and in preventing ciliary clearance, they also affect the in vivo toxicity of nanoparticles (Table 3) [128]. In studies focused on microparticle dynamics, it has been observed that particles exceeding 5 µm in diameter are typically cleared by ciliary action and mucus within the oropharynx and nasal cavity. Particles ranging from 3 to 5 µm in size predominantly deposit in the upper respiratory tract, while those measuring between 0.5 and 3 µm are more likely to penetrate into the lower respiratory tract. For effective alveolar deposition, particles with diameters less than 1 µm are most efficacious. Small-sized nanoparticles showed wide distribution in the upper and lower airways [29]. Nanoparticles with an average diameter of 250 nm–270 nm show little or no movement in the sputum of COPD patients, whereas polystyrene nanocarriers with a diameter of 120 nm can move more easily, and macrophages tend to phagocytose particles larger than 100 nm [142]. However, smaller is not always better; nanoparticles are typically designed to be between 10 and 200 nm. Particles smaller than 10 nm may be cleared by the liver and kidneys through the reticuloendothelial system [119]. Besides, the rapid clearance by cilia limits the contact time between drugs and the mucosa, thereby affecting absorption efficiency. Due to ciliary motion, both 60 nm and 125 nm PLGA nanoparticles can be internalized into nasal tissues within 30 min; however, their total absorption accounts for less than 5% of the available nanoparticles. Additionally, larger particles (125 nm) have higher loading capacities and are present in the submucosal tissues at twice the amount of 60 nm particles [143]. The shape of nanoparticles also affects their cellular uptake. When compared to spherical particles, flat-shaped particles have better sedimentation and migration capabilities, which allow them to more effectively reach deep bronchial ends. Rod-shaped cationic nanoparticles are more readily internalized by cells compared to other shapes, possibly because they are recognized by immune cells as rod-shaped bacteria [144]. Furthermore, studies have indicated that the shape and surface charge of nanoparticles affect their circulatory half-life [145]. Neutral and negatively charged nanoparticles have been shown to reduce serum protein adsorption, thereby extending their circulatory half-life. However, at inflammatory sites, positively charged nanoparticles are more readily bound and internalized by cells. Moreover, positively charged nanoparticles can escape endosomes via the “proton sponge” effect, and thereby avoid degradation within endosomes [146]. Researchers have resolved this contradiction with a clever bipolar switchable charge design, which may play a significant role in future airway drug delivery systems [147, 148].

**Table 3:** The effect of nanoparticles’ physical properties on drug delivery

Physical Property	Types	Advantages	Disadvantages
Size	< 10 nm	• Ease of cell membrane penetration • Increased bioavailability	• Potential for rapid clearance • Possibility of toxic reactions
	10–200 nm	• Increased bioavailability • Enhanced drug release control	• Need for precise control • Risk of aggregation
	> 250 nm	• Increased drug stability	• Difficulty in penetrating mucosal barriers • Potential for uneven drug distribution
Shape	Spherical	• Good circulation time • Ease of manufacturing and surface modification	• May not be suitable for specific targeting • Limited cellular uptake efficiency
	Rod-like	• Enhanced cellular interaction • Increased cellular internalization efficiency	• Complex manufacturing process • Tendency to aggregate, affecting in vivo distribution
	Tabular	• Larger surface area-to-volume ratio • Better deposition and migration ability	• Tendency to aggregate, affecting in vivo distribution • Potential for rapid clearance
	Other shapes	• Designable specific functions • Increased targeted delivery capability	• Complex manufacturing process • Higher production costs
Charge	Positive	• Enhanced cellular uptake • Enhanced interaction with cell membranes	• Potential to trigger immune responses • Possible cytotoxicity
	Negative	• Reduced immune clearance • Improved drug Stability	• Lower cellular uptake efficiency • Possible impact on Drug release
	Neutral	• Good biocompatibility • Lower immune response	• Lower cellular uptake efficiency • Affect targeted delivery capacity

### Challenges of Clinical Translation

#### Toxicity

When designing effective airway drug formulations, the risk of toxicity is not only associated with the drug itself but also with active ingredients and excipients. Minimizing both local and systemic toxicity is crucial. Although inflammatory airway diseases are typically treated with local administration, thereby bypassing first-pass metabolism, the inherent biotoxicity of nanoparticles, especially those that are difficult to degrade, is unavoidable. The accumulation of nanotherapeutics in non-targeted tissues and organs poses long-term toxicity issues. For example, liposomal nanotherapeutics of size 30–200 nm on intravenous administration may accumulate to organs like liver, spleen, kidneys, and heart, which can lead to low therapeutic efficiency and significant systemic side effects [149]. Additionally, drugs can have local side effects on the airways. Some drugs, like decongestants (e.g., corticosteroids, oxymetazoline) and those that increase blood flow (e.g., salbutamol, isoproterenol), can affect blood flow and potentially lead to nasal bleeding [150, 151]. This should be considered when using nano-delivery systems. The average physiological pH of the airway mucosal surface is maintained at 6.3, which is slightly acidic, ensuring proper ciliary clearance function. Therefore, nano-drugs should maintain a pH between 4.5 and 6.5 to avoid airway irritation [152]. Based on the airway microenvironment, researchers have designed drug delivery systems that respond to local stimuli (e.g., pH, temperature, enzyme activity, or ROS levels) can control drug release more precisely, target the diseased site more accurately, and reduce off-target toxicity [153, 154]. It is also essential to ensure that the drug is free from microbial contamination and degradation. Therefore, the potential for preservatives and enhancers to cause irritation and toxicity to the airways must be thoroughly considered by developers. The potential toxicity risks are summarized in Table 4 and provided corresponding mitigation strategies.

**Table 4:** Potential toxicity of nano-delivery systems in the airway and mitigation strategies

Toxicity	Strategies	References
Accumulation of certain drugs	• Use safer nano-carriers • Reduce drug toxicity • Enhance drug metabolism	[149–151]
Local irritation	• Control the dosage of administration • Adapt to the airway microenvironment	[152]
Microbial contamination	• Use safe preservatives and enhancers	[153, 154]

#### Suitable Animal Models

Animal models are crucial for evaluating the pharmacokinetics and potential toxicity of inhaled nano-delivery systems. Although most preclinical in vivo studies have used rodents as the primary model, due to their low cost, ease of handling, and favorable genetic and immunological characteristics. Studies have shown that the airways of rodents differ from those of humans, rodents have narrower nasal cavities, shorter and wider tracheas compared to humans, and simpler lung lobe structures with only two lobes [128]. This might explain differences in nanoparticle clearance and translocation rates [155, 156]. Pigs or non-human primates, whose lungs more closely resemble human lungs, are more suitable for in vivo aerosol deposition studies [157, 158]. In intranasal vaccination studies, non-human primates are considered the best predictive models due to their anatomical, immunological, and nasal microbiome similarities to humans. Additionally, sheep, pigs, and cattle are promising and cost-effective models for intranasal vaccination research [159, 160]. We have summarized the characteristics of commonly used species in airway disease research in Table 5. However, despite the positive and promising preclinical results obtained when using in vivo animal models to deliver nanomedicines to the lungs (including diagnostic and therapeutic agents), the translation from animals to humans remains challenging and requires further methodological validation steps, the physiological conditions of the airway, immune mechanisms, delivery routes, and devices should be further considered, as these factors will limit translation to humans.

**Table 5:** Characteristics of common animal models in airway disease research

Species	Advantages	Disadvantages
Rodents	• Low cost • Easy to handle • Rapid breeding • Similar genetic background	• Narrower nasal cavity • Differences in lower airway structure • Variations in the immune system [160]
Non-human primate	• Similar airway structures • Comparable immune systems	• Ethical concerns • High costs • Difficulty in acquisition
Pig	• Similar airway structures • Strong stress resistance • Ease of handling	• Variations in the immune system [160]

### Large-Scale Production

Nano-delivery systems have been widely used in drug delivery and imaging. However, these delivery systems can be difficult to synthesize, which can lead to their experimental failure. Even when successful in basic research, some nanomedicines cannot be used clinically. This is because it remains challenging to repeatedly manufacture large batches of nanoparticles with consistent properties. During large-scale production, inconsistencies in structure/shape, unclear surface chemistry, and changes in drug loading and delivery processes can occur, and any of these factors can potentially increase the risk for an undesirable biological distribution of nanoparticles. Consequently, the scale-up production process poses a significant challenge. As the processes have not yet been perfected, quality control is difficult, and scaling up production can be costly, which can make these advanced therapies more expensive than existing treatments [161, 162].

## Conclusion and Future Directions

As a cutting-edge technology, nanomaterials have been extensively researched across various medical fields and played a significant role in the treatment of certain diseases. Nanomaterials can provide new strategies for overcoming challenges in drug delivery and enhance therapeutic efficacy when treating airway inflammatory diseases. This review has summarized the applications of nanomaterials in treatment of airway inflammatory diseases, including their use for delivering traditional drugs to novel molecular targets. Most of these studies are still in the early stages of basic research and have shown promising therapeutic effects.

However, numerous challenges remain for clinical translation, as mentioned towards the end of this article. Optimization of product features such as surface properties, particle size, and shape is crucial. Future efforts should focus on designing nanoparticles that are more suitable for mucus penetration, easily degradable, and capable of targeting multiple sites. This process also places higher demands on the drug’s stability, nebulization performance, particle size distribution, and sustained release characteristics [163]. Additionally, the design of nanocarriers sensitive to physical or chemical properties such as pH and temperature could enable effective drug release at specific sites, reduce systemic toxicity for long-term safety, and improve therapeutic efficacy. Given the increasing prevalence of airway inflammatory diseases, more effective treatment methods are urgently needed. The rapid development of smart nano-drug delivery systems, especially when combined with artificial intelligence (AI) and personalized medicine, offers the potential for more precise and effective treatment options for patients [35, 164]. For instance, automated identification and analysis of intracellular nanoparticles, and smart textiles for real-time monitoring of physiological parameters with data analysis via AI algorithms [165, 166], are promising advancements. However, we must also consider the potential risks of nanomaterials entering aquatic environments during production and use, posing threats to aquatic life, ecosystems, and human health. Additionally, the health risk assessment of nanomedicines in human applications is critical. The US Food and Drug Administration (FDA) has implemented specific review and regulatory measures for nanoproducts (https://www.fda.gov/drugs). This review will help researchers understand the challenges and consistencies in airway drug delivery and nanocarrier design and thereby advance the development of new nanomaterial-based drug delivery systems to address multidrug resistance, improve targeting efficiency to organs, and reduce adverse effects.

## Data Availability

No datasets were generated or analysed during the current study.
